# Supplementary material for: Cloud-based Electronic Health Records for Real-time, Region-specific Influenza Surveillance
Source: Sci Rep. 2016 May 11;6:25732. doi: 10.1038/srep25732 (PMC4863169; doi:10.1038/srep25732)
Supplement: Supplementary Information [file srep25732-s1.docx]

**Cloud-based Electronic Health Records for Real-time, Region-specific Influenza Surveillance.**

**Authors:** M. Santillana^1,2,3^, A. T. Nguyen^3^, T. Louie^4^, A. Zink^5^, J. Gray^5^, I. Sung, J. S. Brownstein^1,2^.

**Author Affiliations:**

^1^ Computational Health Informatics Program, Boston Children’s Hospital, Boston, MA

^2^ Harvard Medical School, Boston, MA

^3^ Harvard School of Engineering and Applied Sciences, Cambridge, MA

^4^ Harvard School of Public Health, Boston, MA

^5^ athenaResearch at athenahealth, Watertown, MA

**Supplementary Material**

The US Department of Health and Human Services (HHS) divides the US into the following 10 regions:

Region 1: *Connecticut, Maine, Massachusetts, New Hampshire, Rhode Island, and Vermont*

Region 2: *New Jersey, New York, Puerto Rico, and the U.S. Virgin Islands*

Region 3: *Delaware, District of Columbia, Maryland, Pennsylvania, Virginia, and West Virginia*

Region 4: *Alabama, Florida, Georgia, Kentucky, Mississippi, North Carolina, South Carolina, and Tennessee*

Region 5: *Illinois, Indiana, Michigan, Minnesota, Ohio, and Wisconsin*

Region 6: *Arkansas, Louisiana, New Mexico, Oklahoma, and Texas*

Region 7: *Iowa, Kansas, Missouri, and Nebraska*

Region 8: *Colorado, Montana, North Dakota, South Dakota, Utah, and Wyoming*

Region 9: *Arizona, California, Hawaii, and Nevada*

Region 10: *Alaska, Idaho, Oregon, and Washington*

**Supplementary Figure**

**Figure 1S.** Values of the linear coefficients associated with each independent variable as a function of time for the national and the 10 regional prediction models.
